# What is the optimal lipid emulsion for preventing intestinal failure-associated liver disease following parenteral feeding in a rat model of short-bowel syndrome?

**DOI:** 10.1007/s00383-020-04802-0

**Published:** 2021-01-03

**Authors:** Seiro Machigashira, Tatsuru Kaji, Shun Onishi, Keisuke Yano, Toshio Harumatsu, Koji Yamada, Waka Yamada, Makoto Matsukubo, Mitsuru Muto, Satoshi Ieiri

**Affiliations:** 1 Department of Pediatric Surgery, Research Field in Medicine and Health Sciences, Medical and Dental Sciences Area, Research and Education Assembly, Kagoshima University, 8-35-1, Sakuragaoka, Kagoshima, 890-8520 Japan; 2 Clinical Training Center, Kagoshima University Hospital, Kagoshima, Japan

**Keywords:** Short-bowel syndrome, Intestinal failure-associated liver disease, Fish oil, Composite lipid emulsion

## Abstract

**Purpose:**

Composite lipid emulsion (CLE) has been used for intestinal failure-associated liver disease (IFALD) to compensate for the disadvantages of soybean oil lipid emulsion (SOLE) or fish oil lipid emulsion (FOLE). However, the influence of its administration is unclear. We evaluated the effects of these emulsions on IFALD using a rat model of the short-bowel syndrome.

**Methods:**

We performed jugular vein catheterization and 90% small bowel resection in Sprague–Dawley rats and divided them into four groups: control (C group), regular chow with intravenous administration of saline; and total parenteral nutrition co-infused with SOLE (SOLE group), CLE (CLE group) or FOLE (FOLE group).

**Results:**

Histologically, obvious hepatic steatosis was observed in the SOLE and CLE groups but not the FOLE group. The liver injury grade of the steatosis and ballooning in the FOLE group was significantly better than in the SOLE group (*p* < 0.05). The TNF-α levels in the liver in the FOLE group were significantly lower than in the SOLE group (*p* < 0.05). Essential fatty acid deficiency (EFAD) was not observed in any group.

**Conclusion:**

Fish oil lipid emulsion attenuated hepatic steatosis without EFAD, while CLE induced moderate hepatic steatosis. The administration of CLE requires careful observation to prevent PN-induced hepatic steatosis.

## Introduction

The survival prognosis of patients with intestinal failures, such as short-bowel syndrome (SBS), intestinal motility disorder, and inflammatory bowel disease, has dramatically improved due to the development of total parenteral nutrition (TPN). Many patients with intestinal failure require long-term PN, which can cause complications, such as catheter-related blood stream infection (CRBSI) and intestinal failure-associated liver disease (IFALD). Kelly et al. stated that IFALD is characterized by a progressive pathology of cholestasis and steatosis inducing hepatic fibrosis and ultimately leading to liver cirrhosis [1]. Cholestasis occurs in 15–80% of IFALD neonates and infants, and steatosis, also known as non-alcoholic fatty liver disease (NAFLD), occurs in 40–60% of IFALD adults.

Several studies have implicated soybean oil lipid emulsion (SOLE) in the occurrence of hepatic cholestasis and steatosis of IFALD for two reasons. First, SOLE is rich in ω-6 polyunsaturated fatty acids (PUFAs), such as linoleic acid. Arachidonic acid, a metabolite of linoleic acid, is a precursor of inflammatory cytokines. Second, phytosterols contained in SOLE may exacerbate the development of cholestasis through the inhibition of bile acid transporters.

Fish oil lipid emulsion (FOLE) is rich in ω-3 PUFAs, such as eicosapentaenoic acid (EPA) and docosahexaenoic acid (DHA). EPA is a substrate of precursors with anti-inflammatory effects. Since the ω-6 PUFA linoleic acid and the ω-3 PUFA α-linolenic acid are metabolized by the same enzymes, the metabolisms of these molecules compete. Furthermore, since FOLE contains less phytosterol than SOLE, it has been used to prevent and treat IFALD [2, 3].

However, as FOLE contains an insufficient amount of ω-6 PUFAs for nutritional needs, concerns about essential fatty acid deficiency (EFAD) and growth failure, especially for children, have arisen. Composite lipid emulsion (CLE), also known as SMOFlipid^Ⓡ^ (Fresenius Kabi Australia Pty Ltd.), is composed of soybean oil (SO) (30%), medium-chain triglycerides (30%), olive oil (25%), and fish oil (FO) (15%). It may be a better lipid emulsion than SOLE and was recently developed and used as a first lipid emulsion due to its nutritional benefits. Composite lipid emulsion has features such as a reduced amount of phytosterols and a different fatty acid composition, including EPA and DHA, compared with SOLE. However, while CLE has been used clinically [4], Lee et al. showed that long-term CLE resulted in cholestasis of IFALD, and changing to FOLE monotherapy improved IFALD [5]. Furthermore, dyslipidemia has been reported as an adverse event in children [6]. The effects of CLE and FOLE in SBS patients are thus unclear at present.

No experimental studies so far have evaluated the effect and mechanisms of lipid emulsions for hepatic steatosis of IFALD in a parenterally fed model following massive bowel resection. In addition, pediatric surgeons sometimes clinically encounter IFALD following massive bowel rection and parenteral nutrition.

We, therefore, compared the effect of three different lipid emulsions (SOLE, CLE and FOLE) for inducing hepatic steatosis of IFALD in a parenterally fed rat model of SBS.

## Methods

### Animals

All experimental procedures were approved by the Laboratory Animal Committees of our institution and were performed according to the Guidelines for the Care and Use of Laboratory Animals (Approval number: MD17024).

We used Sprague–Dawley rats (weighing 200–250 g). The rats were individually housed in metabolic cages with free access to standard rat chow and water and acclimatized to their environment for seven days before the experiments. The laboratory environment was maintained at a standardized temperature (23 ± 1 °C) and humidity (50 ± 10%) and a 12-h light–dark cycle.

#### Study design

The rats were fasted overnight before the experiment. They underwent surgical placement of a central venous catheter and 90% small bowel resection and were then randomly divided into the following groups: (1) Oral feeding with normal chow plus continuous infusion of saline, control (C) group; (2) TPN with SOLE (SOLE) group; (3) TPN with CLE (CLE) group; and (4) TPN with FOLE (FOLE) group. We used Intralipos^®^ (Otsuka Pharmaceutical Co., Ltd. Japan) as the SOLE, SMOFlipid^®^ (Fresenius Kabi Australia Pty Ltd.) as the CLE and Omegaven^®^ (Fresenius Kabi Deutschland GmbH) as the FOLE.

On the 13th day after surgery, the rats were anaesthetized and euthanized by collecting a blood sample via pericardial puncture, and laparotomy was performed for liver sample harvesting. The blood samples were immediately centrifuged at 1500*g* for 15 min at 4 ℃, plasma and serum were extracted, and liver samples were flash-frozen in liquid nitrogen; these samples were stored in −80 °C. Additional liver samples were fixed with 10% formaldehyde for histological analyses. Paraffin sections of formalin-fixed tissue were cut at a thickness of 3 µm for staining with hematoxylin and eosin (H&E) and oil red O.

#### Surgical procedure and maintenance methods

The rats were anesthetized with isoflurane (1.5% inhalation by mask), and cefazolin (50 mg/kg, subcutaneously; Otsuka Pharmaceutical Factory, Inc., Tokushima, Japan) and buprenorphine (0.01 mg/kg, subcutaneously; Otsuka Pharmaceutical Co., Ltd.) were administered. The central venous catheter, a silastic catheter with an outside diameter of 1.2 mm (NIPRO Co., Ltd., Osaka, Japan), was inserted into the jugular vein.

Regarding the surgical procedure, in brief, the jejunum 5 cm distal from the ligament of Treitz and the ileum 5 cm proximal to the ileocecal valve were dissected, and end-to-end anastomosis was performed. After the operation, rats were maintained at 60 ml/day on a low-concentration TPN solution (NEOPAREN^®^ No. 2; Otsuka Pharmaceutical Co., Ltd.) supplemented with a lipid emulsion. The composition of the low-concentration TPN solution was as follows (%): amino acids 2.5, glucose 14.5, and lipids 3.33. After 24 h, the composition of the high-concentration TPN solution was switched to (%): amino acids 3.16, glucose 20.3 and lipids 3.33. The TPN solution provided equivalent nutrients to all TPN-fed animals at 76.4 kcal/rat/day (1.9 g protein, 2.0 g fat and 12.2 g carbohydrate). The rats in the C group were fed 23 g of regular rat chow per day (79 kcal/rat/day), which was almost equal to the calories in the TPN solution.

#### The biochemical examination of the liver function

In serum biochemical tests, aspartate aminotransferase (AST), alanine aminotransferase (ALT), total bilirubin (T-Bil), direct bilirubin (D-Bil), total cholesterol (T-CHO) and triglyceride (TG) were measured using the Japan Society of Clinical Chemistry standardized matching method. These measurements were performed at the Kagoshima Lab of SRL, Inc., Japan, using the 7180 clinical Analyzer (Hitachi High-tech GLOBAL, Japan).

#### Analyses of phytosterols in serum

The phytosterols were measured following Matthan’s paper [7]. Analyses of β-sitosterol and campesterol, samples were saponified to extract sterol components and analysed by gas chromatography. The findings were then compared with the analytical results of the standard product, and the target sterol concentrations were calculated from the peak elution position and area. These analyses were carried out at Skylight Biotech Inc., Akita, Japan.

#### Histological analyses and liver injury grading

The severity was assessed as the grade according to the NAFLD scoring system reported by Nabeshima et al. [8] as follows: steatosis grading [no lipid droplets (normal N); lipid droplets in < 33% of the hepatocytes (low L); lipid droplets in 33–66% of the hepatocytes (moderate M); and lipid droplets in > 66% of the hepatocytes (severe S)], inflammation grading [no inflammation (normal N); < 10 inflammatory foci, each consisting of > 5 inflammatory cells (low L); > 10 inflammatory foci (moderate M); or uncountable diffuse or fused inflammatory foci (severe S)]; and ballooning grading [none (normal N); few balloon cells (low L) or many balloon cells/prominent ballooning (moderate M)]. The histological evaluation of the liver was performed by two blinded pathologists.

#### Analyses of the lipid content of liver tissue and fatty acid composition in serum

According to the FOLCH methods [9], each snap-frozen tissue specimen was homogenised and extracted using chloroform–methanol, and T-CHO, TG and free cholesterol (FC) were measured at Skylight Biotech Inc. The fatty acid composition of the serum samples was assessed. All measurements were performed at Nagahama Life Science Laboratory (ORIENTAL YEAST CO., LTD., Shiga, Japan).

#### Quantitative real-time polymerase chain reaction (qPCR) of cytokines in the liver tissue

We evaluated the interleukin-6 (IL-6) and tumor necrosis factor-α (TNF- α) mRNA expressions in liver tissue. The procedure was used superscript 77IV reverse transcriptase (Thermo Fisher Scientific, Waltham, MA, USA) with oligo (dT) primer, RPr IL-6 F1: 5′-GTATGAACAGCGATGATGATGCACTGTC-3′ (forward), RPr IL-6 R1: 5′-TAGAAACGGAACTCCAGAAGACCAG-3′ (reverse), RPr TNF-α F1: 5′-ACGCTCTTCTGTCTACGAACTTCG-3′ (forward) and RPr TNF-α R1: 5′-AAGATGATCTGAGTGAGGGTCTG-3′ (reverse), and the expression of each gene was analyzed using the Bio-Rad CFX96 system (Bio-Rad Laboratories, Inc., Hercules, CA, USA). These measurements were performed by Repertoire Genesis Inc., Osaka, Japan.

#### Analyses of the expression of Rubicon antibody

We assessed the expression of Rubicon antibody, a protein that suppresses autophagy, using Western blotting. GTX 31,593 Rubicon antibody from GeneTex, Inc., was used as the primary antibody. Western blotting was performed by IDEA Consultants, Inc., Tokyo, Japan.

#### Statistical analyses

All statistical analyses were performed with Easy R (Saitama Medical Center, Jichi Medical University, Tochigi, Japan), which is a graphical user interface for R (The R Foundation for Statistical Computing, version 3.3.2). The statistical analyses of the data were performed using a one-way analysis of variance (ANOVA) followed by the Tukey–Kramer and Kruskal–Wallis test and the Steel–Dwass multiple comparisons post hoc test. *P* values of < 0.05 were considered to indicate statistical significance.

## Results

### Serum biochemical examinations of the liver function

The serum ALT levels were significantly higher in the C group than in the SOLE group (*p* < 0.05) (Table 1). The serum T-CHO levels were significantly higher in the SOLE group than in the FOLE group (*p* < 0.05). There were no significant differences in any other evaluation endpoints, including bilirubin, among the four groups.

**Table 1:** Serum biochemical examination findings

	(Mean range)	C group	SOLE group	CLE group	FOLE group
AST (IU/L)	(117.3–166.2)	179.36 ± 76.09	142.11 ± 31.27	190.00 ± 95.99	122.50 ± 35.45
ALT (IU/L)	(20.0–23.8)	36.75 ± 7.92^†^	22.78 ± 6.56	26.50 ± 10.41	26.60 ± 9.71
T-Bil (mg/dl)	(0.07–0.09)	0.10 ± 0.07	0.10 ± 0.87	0.19 ± 0.08	0.13 ± 0.08
D-Bil (mg/dl)	–	0.03 ± 0.05	0.04 ± 0.06	0.09 ± 0.05	0.05 ± 0.06
T-CHO (mg/dl)	(70.4–75.9)	50.13 ± 11.29	63.89 ± 22.66^#^	54.88 ± 10.60	43.30 ± 16.96
TG (mg/dl)	(29.6–53.5)	17.50 ± 18.84	42.89 ± 22.71	35.00 ± 24.80	34.80 ± 31.20

The values are presented as the mean ± standard deviation

*AST* aspartate aminotransferase, *ALT* alanine aminotransferase, *T-Bil* total bilirubin, *D-Bil* direct bilirubin, *T-CHO* total cholesterol, *TG* triglyceride

^†^*p* < 0.05 vs. SOLE group

^#^*p* < 0.05 vs. FOLE group

#### Histological analyses of the liver

**Fig. 1:**
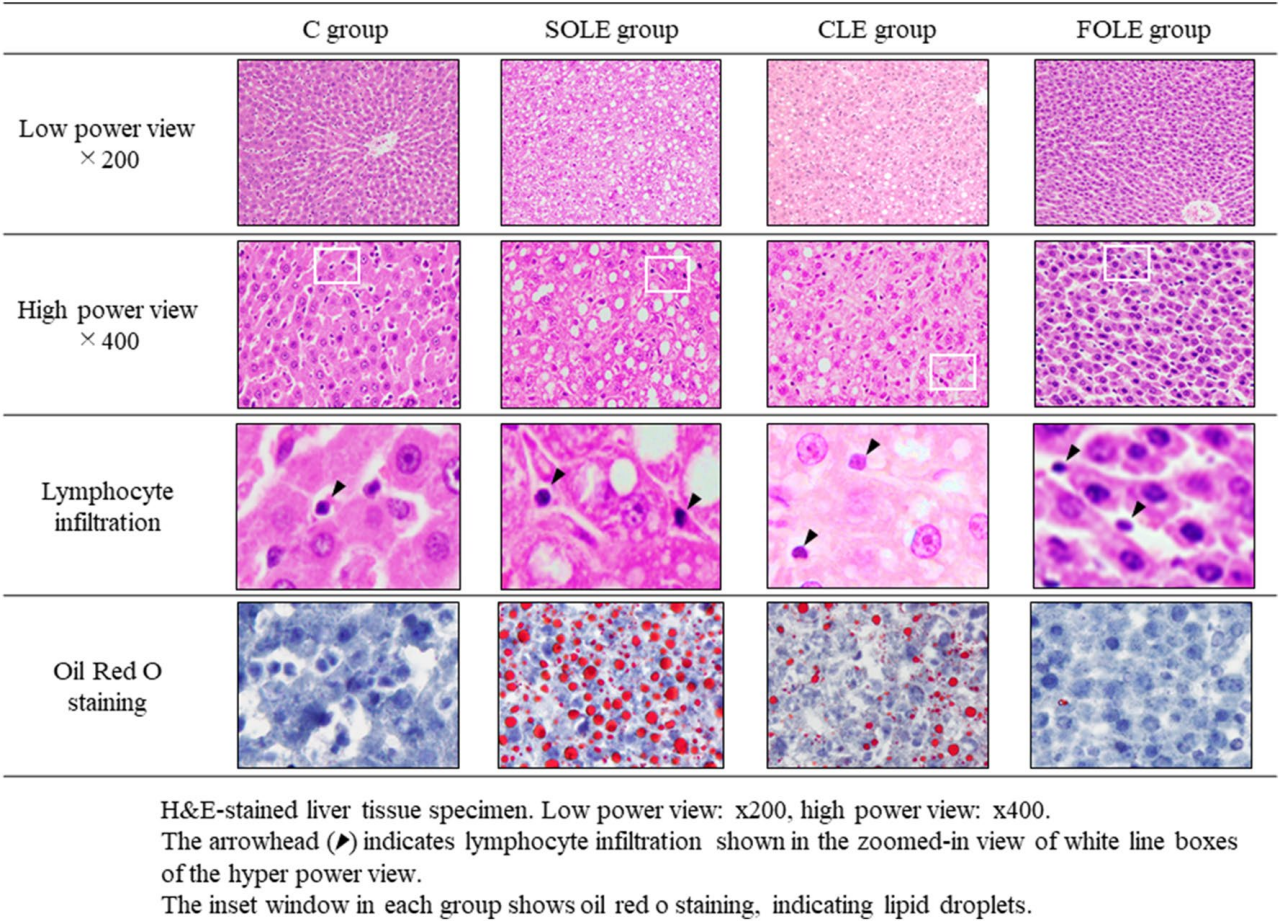
A histological analysis of the liver tissue specimens. H&E and oil red O staining of the liver tissue specimen. Low-power view: × 200, high power view: × 400. The arrowhead indicates lymphocyte infiltration shown in the zoomed-in view of white line boxes of the high power view

HE staining and oil red O staining of liver sections is shown in Fig. 1. Representative histology of the C group showed no lipid droplets or inflammatory cell infiltration. In contrast, histology of the SOLE group showed both inflammatory cells and lipid droplets. In this specimen, diffuse moderate-to-severe steatosis was observed. Even in the histology of the CLE group, lipid droplets were observed, although not as severe as in the SOLE group. In the FOLE group, lipid droplets were scarcely recognized, findings that were similar to the C group.

**Fig. 2:**
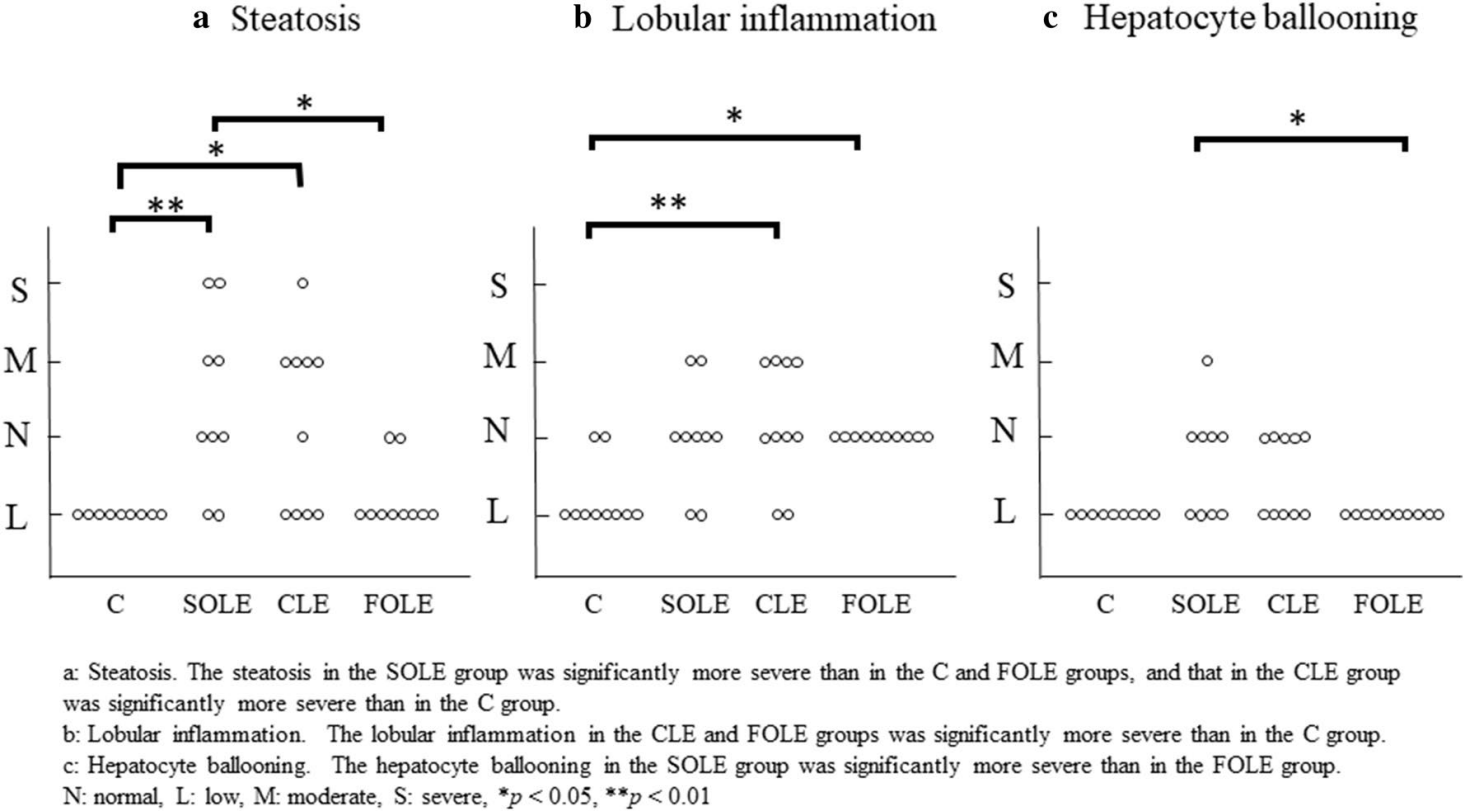
Liver injury grade. **a** Steatosis. The steatosis in the SOLE group was significantly more severe than in the C and FOLE groups, and that in the CLE group was significantly more severe than in the C group. **b** Lobular inflammation. The lobular inflammation in the CLE and FOLE groups was significantly more severe than in the C group. **c** Hepatocyte ballooning. The hepatocyte ballooning in the SOLE group was significantly more severe than in the FOLE group. *N* normal, *L* low, *M* moderate, *S* severe, *p < 0.05, **p < 0.01

We also evaluated the grade of liver injury using the NAFLD activity score reported by Nabeshima et al. The grades of liver injury included the following three points: steatosis, lobular inflammation, and hepatocyte ballooning (Fig. 2). The steatosis in the SOLE group was significantly more severe than in the C and FOLE groups [vs. C (*p* < 0.01), vs. FOLE (*p* < 0.05)]. Furthermore, the steatosis in the CLE group was significantly more severe than in the C group [vs. C (*p* < 0.05)] (Fig. 2a). The lobular inflammation in the CLE and FOLE groups were significantly more severe than in the C group [vs. FOLE (*p* < 0.05), vs. CLE (*p* < 0.01)] (Fig. 2b). The moderate grade injury of the lobular inflammation was observed only in the SOLE and CLE groups. The hepatocyte ballooning in the SOLE group was significantly more severe than in the FOLE group [vs. FOLE (*p* < 0.05)] (Fig. 2c).

#### Analyses of the lipid content in liver tissue

There were no significant differences in the T-CHO or FC level in the liver among groups. However, the TG level in the liver tissue in the SOLE group was significantly higher than in the control and FOLE groups [C 1.31 ± 1.21 mg/g, SOLE 29.37 ± 28.21 mg/g, CLE 19.88 ± 14.38 mg/g, FOLE 3.88 ± 1.41 mg/g; SOLE vs. C, FOLE (*p* < 0.05)].

#### Fatty acid analyses of serum

The fatty acid profile of the serum, including the triene-to-tetraene (TT) ratio, is shown in Table 2. The fatty acid profile tended to depend mostly on the composition of the administered lipid emulsion. The serum levels of linoleic acid in the SOLE and CLE groups were significantly higher than in the FOLE group (*p* < 0.01). Arachidonic acid levels in the SOLE and CLE groups were also significantly higher than in the FOLE group (vs. SOLE; *p* < 0.01, vs. CLE; *p* < 0.05), with a significant difference between the SOLE and CLE groups as well (*p* < 0.01). The serum levels of EPA in the FOLE and CLE groups were significantly higher than in the SOLE group (*p* < 0.01), with a significant difference between the FOLE and CLE groups as well (*p* < 0.05). The serum α-linolenic acid levels in the SOLE group were significantly higher than in the FOLE group (*p* < 0.01). The serum oleic acid levels in the CLE group were significantly higher than in the SOLE and FOLE groups (*p* < 0.01).

**Table 2:** Fatty acid composition of serum and the TT ratio

		C group	SOLE group	CLE group	FOLE group
ω-3	α-Linolenic acid (C18:3)	2.61 ± 2.43	6.90 ± 2.48** ^##^	5.23 ± 3.68	2.68 ± 1.21
	EPA (C20:5)	12.73 ± 7.34	21.89 ± 9.88	75.16 ± 14.51**^††^	129.2 ± 43.79**^†† §^
	DHA (C22:6)	62.41 ± 22.67	99.90 ± 40.88	132.7 ± 18.76**	155.8 ± 51.09**
ω-6	Linoleic acid (C18:2)	191.1 ± 93.02^##^	286.9 ± 49.12*^##^	218.3 ± 56.61^##^	76.85 ± 33.71
	Arachidonic acid (C20:4)	257.8 ± 69.59^##^	358.0 ± 107.0*^§§##^	174.06 ± 33.09^#^	79.94 ± 21.27
ω-9	Oleic acid (C18:1)	72.83 ± 50.15	101.3 ± 28.78	182.1 ± 74.19*^††##^	91.78 ± 41.69
	Mead acid (C20:3)	0.94 ± 0.46	1.51 ± 0.56^##^	1.40 ± 0.30^##^	0.71 ± 0.23
TT ratio		0.0037 ± 0.0018	0.0044 ± 0.0023	0.0081 ± 0.0017*	0.0091 ± 0.0023*^†^

All values are shown in units of μg/ml

*EPA* eicosapentaenoic acid, *DHA* docosahexaenoic acid, *TT* ratio triene-to-tetraene ratio

*p < 0.05 vs. C; **p < 0.01 vs C; ^†^*p* < 0.05 vs. SOLE ^††^*p* < 0.01 vs. SOLE

^§^*p* < 0.05 vs. CLE; ^§§^*p* < 0.01 vs. CLE; ^#^*p* < 0.05 vs. FOLE; ^##^*p* < 0.01 vs. FOLE

The ratio of eicosatrienoic acid (mead acid) to eicosatetraenoic acid (arachidonic acid), called the TT ratio, was used to diagnose EFAD (TT ratio > 0.2, according to previous reports [10]). The TT ratio in the FOLE group was significantly higher than in the C and SOLE groups (*p* < 0.05), and that in the CLE group was higher than in the C group (*p* < 0.05). However, the TT ratios in the CLE and FOLE groups were both lower than 0.2.

#### Analyses of phytosterols in serum

**Fig. 3:**
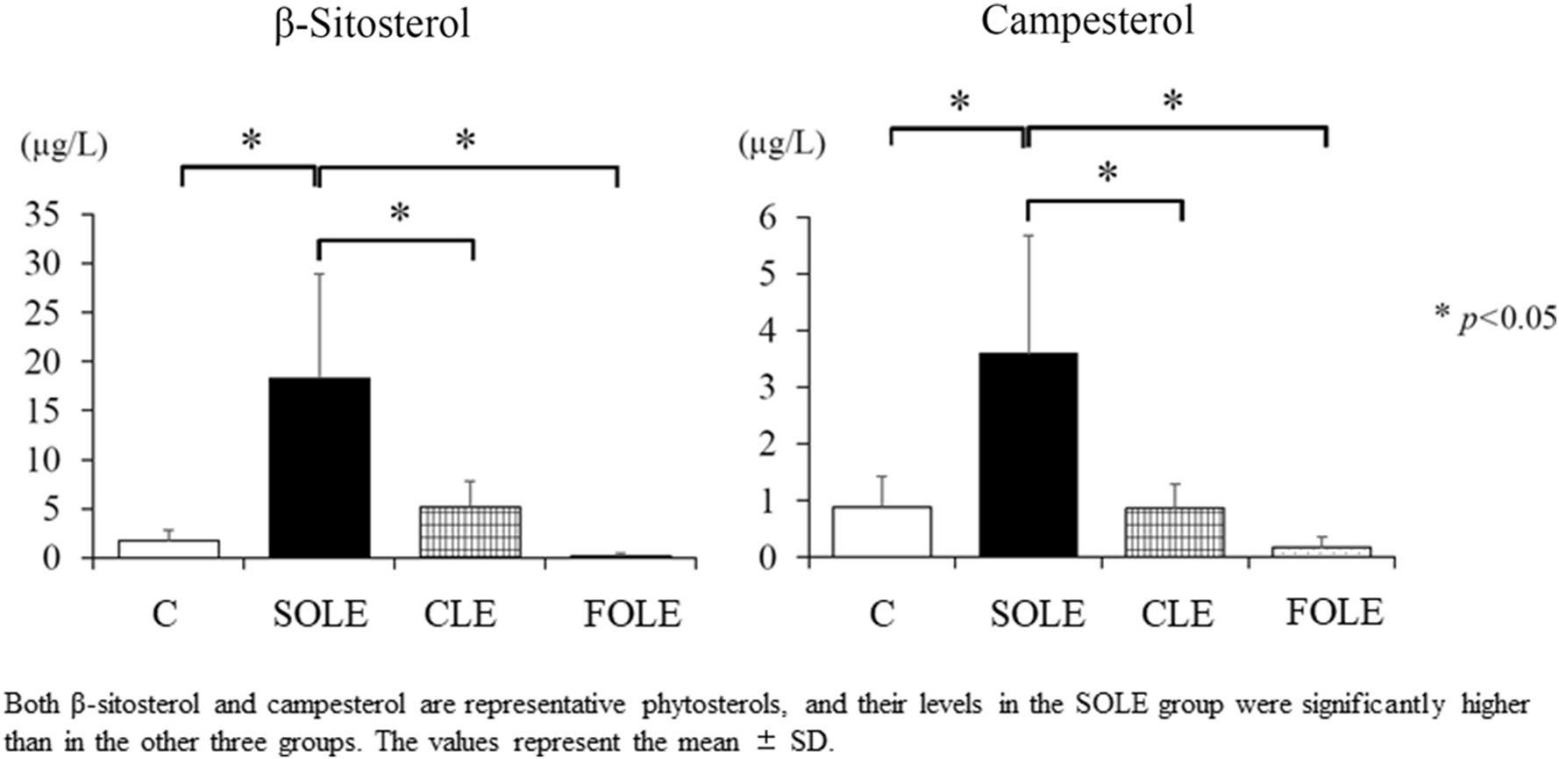
The analysis of phytosterols in serum. Both β-sitosterol and campesterol levels in the SOLE group were significantly higher than in the other three groups. The values represent the mean ± SD. *p < 0.05

The serum β-sitosterol levels in the SOLE group were significantly higher than in the other 3 groups (*p* < 0.05) (Fig. 3), with similar results found for campesterol.

#### Analyses of inflammatory cytokines in liver tissue

**Fig. 4:**
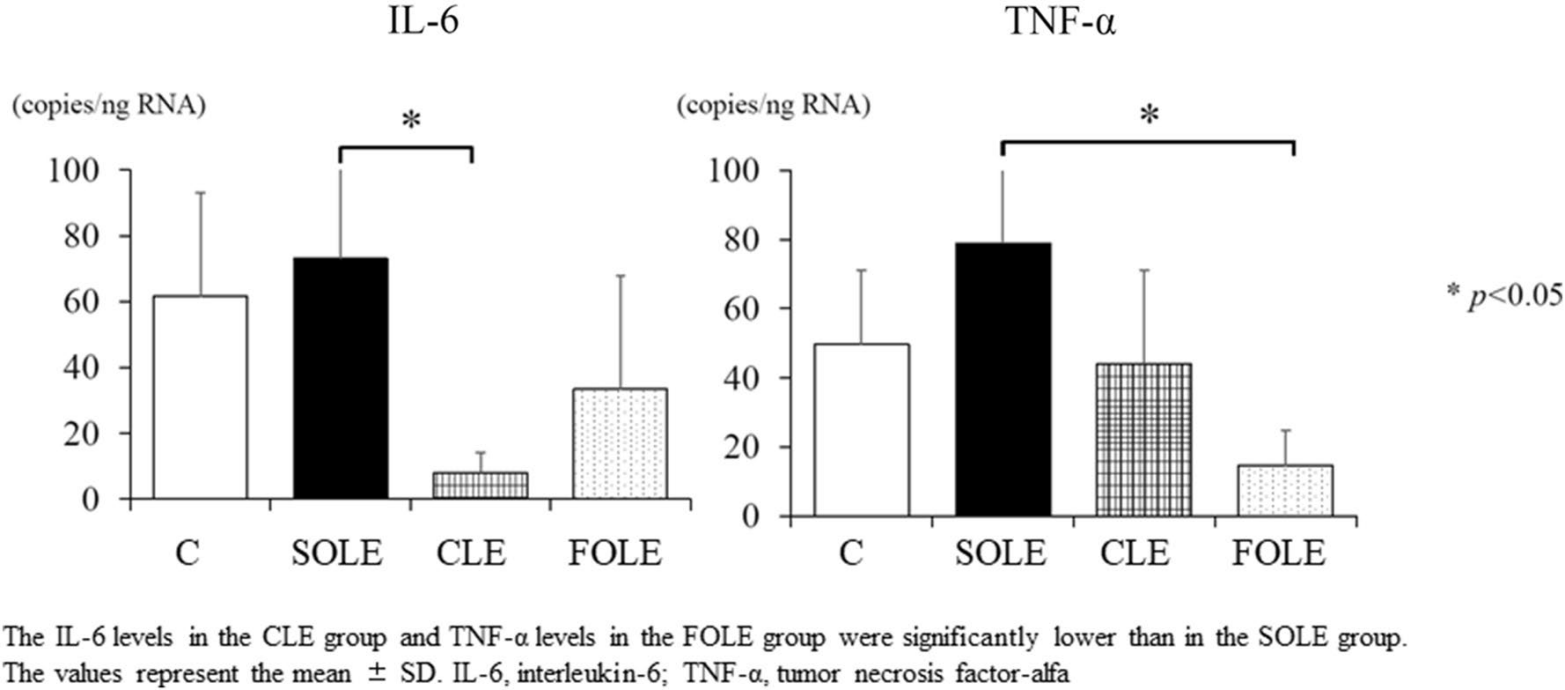
Inflammatory cytokines in the liver tissue. The IL-6 levels in the CLE group and TNF-α levels in the FOLE group were significantly lower than in the SOLE group. The values represent the mean ± SD. IL-6, interleukin-6; TNF-α, tumor necrosis factor-alfa. *p < 0.05

The inflammatory cytokine levels in the liver tissue are shown in Fig. 4. The IL-6 levels in the CLE group were significantly lower than in the SOLE group (*p* < 0.05), and the TNF-α levels in the FOLE group were significantly lower than in the SOLE group (*p* < 0.05).

#### The expression of Rubicon antibody in hepatocytes in the Western blotting analysis

**Fig.5:**
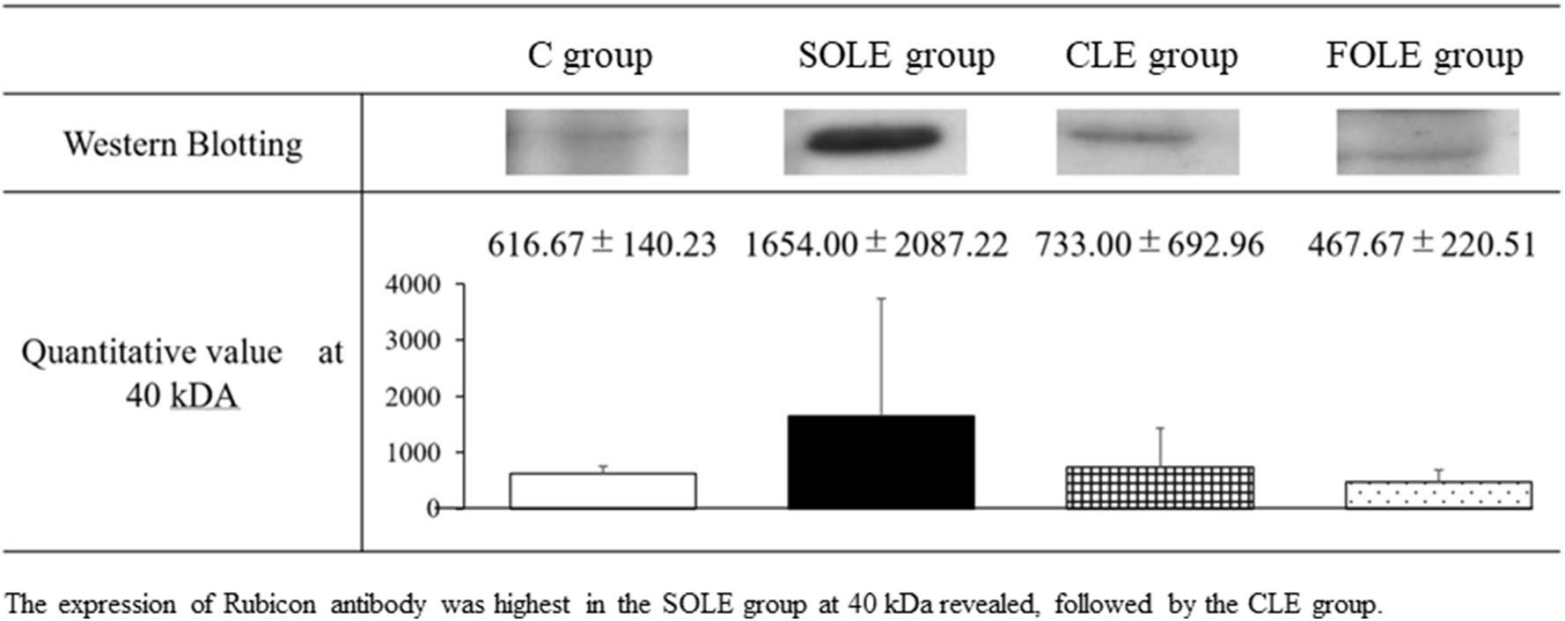
The expression of Rubicon antibody in hepatocytes in the Western blotting analysis. The expression of Rubicon antibody was highest in the SOLE group at 40 kDa revealed, followed by the CLE group

The expression of Rubicon antibody was most enhanced in the SOLE group, followed by the CLE group (Fig. 5). The quantitative values showed a similar tendency to the histological findings of hepatic steatosis.

## Discussion

Patients with intestinal failure who require long-term PN are at risk of developing IFALD. Lauriti et al. reported that 50% of patients undergoing long-term PN were afflicted with this disease [11]. IFALD is associated with cholestasis and steatosis and progresses to liver cirrhosis and liver failure. Among potential risk factors, the intravenous administration of SOLE may be involved in the development of IFALD. Several studies have shown that optimal lipid emulsion might improve the prognosis of SBS patients [2, 12, 13]. We conducted our study to compare the efficacy of three lipid emulsions for hepatic steatosis of IFALD using a parenterally fed rat model following massive bowel resection.

The major findings of the current study were as follows: (1) the serum ALT level was not associated with histological hepatic steatosis; (2) the serum T-CHO levels in the SOLE group were significantly higher than in the FOLE group; (3) the serum β-sitosterol and campesterol levels in the SOLE group were significantly higher than in the other three groups; (4) regarding hepatic histology, SOLE and CLE induced severe and moderate hepatic steatosis, respectively, while FOLE did not steatosis at all; (5) the levels of hepatic inflammatory cytokines, especially TNF-α, were significantly lower in the FOLE group than in the SOLE group; and (6) PN with FOLE did not induce EFAD based on the TT ratio.

The serum ALT level in the control group was significantly higher than in the SOLE group, but this value slightly exceeded the upper limit of the normal range. The most important point of our serum biochemical tests was that the serum ALT level did not reflect the histological severity of hepatic steatosis. The severity of hepatic steatosis was not necessarily associated with the hepatic transaminase level, so we must be alert for hepatic steatosis during PN, even when hepatic transaminase levels are within normal limits clinically.

In our experiments, the serum T-CHO level in the SOLE group was significantly higher than in the FOLE group. A recent clinical study for pediatric patients with IFALD showed that the increase in serum plant sterol levels by SOLE augmented cholesterol synthesis [14]. In our study, the β-sitosterol and campesterol levels in the SOLE group were significantly higher than in the other three groups. The augmentation of plant sterol levels due to SO in the SOLE group may induce significant hypercholesterolemia.

According to the histological liver injury grading, hepatic steatosis in the SOLE groups was significantly more severe than in the C and FOLE groups, and that in the CLE group was also more severe than in the C group. Moderate and severe hepatic steatosis was observed in both the SOLE and CLE group, which included SO. Regarding the lobular inflammatory grading, surprisingly, the CLE and FOLE groups had statistical significance compared with the C group. It should be noted that there was no significant difference between the C and SOLE groups, but instead that the lobular inflammation in the SOLE and CLE groups alone showed moderate grade damage. The hepatocyte ballooning in the SOLE group was significantly more severe than that in the FOLE group, despite there being no significant difference from the C group. The results of hepatocyte ballooning are considered to be statistical interpretations based on differences in sample size. These findings suggest that the histological liver injury caused by TPN in the SBS rat model may be associated with SO. Nandivada et al. previously reported that parenteral SO with parenteral FO, even in a 1:1 ratio, induced hepatic steatosis [15]. Their experimental SO fat dose included 1.2 g/kg/day. In our experiment, the SO fat dose in the CLE group was only 0.6 g/kg/day. Based on these results, even a small amount of SO fat may strongly affect hepatic steatosis. In addition, the findings of lobular inflammation and hepatocyte ballooning were not concomitant with the level of inflammatory cytokines. Hence further studies are required to clarify the relationship between the histological changes, such as lobular inflammation and hepatocyte ballooning, and the cytokine levels.

SOLE supplies abundant ω-6 PUFAs, such as linoleic acid, which is metabolized into arachidonic acid. Arachidonic acid is a substrate of pro-inflammatory eicosanoids. In contrast, FOLE supplies abundant ω-3 PUFAs, such as EPA and DHA. EPA is a substrate of anti-inflammatory eicosanoids. Because ω-3 and ω-6 PUFAs have competing metabolisms. In our study, according to its fatty acid profile, nevertheless SOLE itself did not include arachidonic acid, while the amount of arachidonic acid in the SOLE group was significantly higher than in the other three groups. Since SOLE contained five times linoleic acid of ω-6 PUFAs as much as α-linolenic acid of ω-3 PUFAs, this result implied that SOLE would metabolize ω-6 PUFAs more significantly than ω-3 PUFAs. Several studies have also suggested that hepatic steatosis may be induced by SOLE and attenuated by FOLE [2, 16], with similar results shown here.

In our study, however, moderate steatosis was observed in the CLE group. According to the fatty acid profile, arachidonic acid levels in the CLE group were significantly lower than in the SOLE group but higher than in the FOLE group. Furthermore, EPA and DHA levels in the CLE group were significantly lower than in the FOLE group but higher than in the SOLE group. Given the interaction between ω-6 and ω-3 PUFAs, we speculate that an imbalance of EPA, such as an anti-inflammatory effect, and arachidonic acid, such as a pro-inflammatory effect, may have been associated with the occurrence of hepatic steatosis in the CLE group. The composition of CLE may, therefore, require some modification to prevent hepatic steatosis.

Many studies have explored the relationship between steatosis and inflammatory cytokines. We evaluated hepatic IL-6 and TNF-α levels. Given the relationship between the severity of hepatic steatosis and cytokines, the expression of TNF-α was more concomitant with the severity of hepatic steatosis than that of IL-6. Hotamisligil et al. also stated that TNF-α was a key factor in the development of NAFLD [17]. In the present study, hepatic TNF-α levels were significantly higher in the SOLE group and non-significantly higher in the CLE group than in the FOLE group. Although we speculated that TNF-α in the liver would play a pivotal role in the development of hepatic steatosis in our model, however, we have not measured anti-inflammatory cytokines in this study, and further studies are needed to clarify the mechanism. Our experimental model closely reflects clinical IFALD because of the PN feeding following a massive bowel resection, making it useful for further studies.

Hepatic autophagy is impaired in NAFLD, and Tanaka et al. suggested that Rubicon, an autophagy-regulating protein, played a pathogenetic role in NAFLD by inducing lipid accumulation via the inhibition of autophagy [18]. We, therefore, suspected that autophagy was also related to steatosis in IFALD and analyzed the Rubicon antibody levels using Western blotting. The expression of Rubicon antibody in the SOLE group was highest, and that in the CLE group was higher than in the control and FOLE groups. Given our result, we speculated that Rubicon might be involved in the development of hepatic steatosis in our model. Further studies will be required to clarify this mechanism. If autophagy is indeed related to the onset of IFALD, as with NAFLD, this finding may help establish prevention and therapeutic methods in the future.

The current study showed that FOLE attenuated steatosis, just as was shown in previous reports [13]. However, given that this emulsion contains < 7% linoleic acid, there is a risk of EFDA with the standard dose of FOLE, so manufacturers do not recommend FOLE monotherapy. The TT ratios in the FOLE and CLE groups in our study were increased compared with the C group but still well below 0.2, and clinical symptoms of EFAD, such as hair loss and dry skin, were not observed. Given the TT ratio in our study, FOLE and CLE therapy appear safe and acceptable without risk of EFAD. However, this result is only based on the findings of a 2-week animal experiment, and it is, therefore, necessary to carefully observe the clinical symptoms and measure the fatty acid fraction levels regularly in actual clinical practice.

## Conclusion

Hepatic steatosis of IFALD, known as NAFLD, was observed after the administration of SOLE and CLE using a parenterally fed rat model with SBS, which is an authentic IFALD model, as these surgical and nutritional conditions closely resemble those of clinical cases of SBS. Hepatic steatosis was not observed on the administration of FOLE. Based on the histological findings of hepatic steatosis, FOLE attenuated hepatic steatosis without EFAD and CLE may require modification for IFALD patients with long-term PN. Furthermore, inflammatory cytokines, especially TNF-α, and Rubicon may be associated with the development of hepatic steatosis. Further studies are needed to clarify (1) the mechanism underlying the onset of hepatic steatosis using lipid emulsion with SO and (2) the optimal composition of CLE that will not induce hepatic steatosis.

## Abbreviations

CLE: Composite lipid emulsion
CRBSI: Catheter-related blood stream infection
DHA: Docosahexaenoic acid
EFAD: Essential fatty acid deficiency
EPA: Eicosapentaenoic acid
FO: Fish oil
FOLE: Fish oil lipid emulsion
IFALD: Intestinal failure associated liver disease
NAFLD: Non-alcoholic fatty liver disease
PUFAs: Polyunsaturated fatty acids
SBS: Short bowel syndrome
SO: Soybean oil
SOLE: Soybean oil lipid emulsion
TPN: Total parenteral nutrition
